# Personalized predictive lung dosimetry by technetium-99m macroaggregated albumin SPECT/CT for yttrium-90 radioembolization

**DOI:** 10.1186/s13550-014-0033-7

**Published:** 2014-06-29

**Authors:** Yung Hsiang Kao, Butch M Magsombol, Ying Toh, Kiang Hiong Tay, Pierce KH Chow, Anthony SW Goh, David CE Ng

**Affiliations:** 1 Department of Nuclear Medicine and PET, Singapore General Hospital, Outram Road, Singapore 169608, Singapore; 2 Department of Nuclear Medicine, Austin Hospital, Level 1, Harold Stokes Building, 145 Studley Rd, Melbourne 3084, Victoria, Australia; 3 Department of Diagnostic Radiology, Singapore General Hospital, Outram Road, Singapore 169608, Singapore; 4 Department of General Surgery, Singapore General Hospital, Outram Road, Singapore 169608, Singapore; 5 Department of Surgical Oncology, National Cancer Centre Singapore, 11 Hospital Drive, Singapore 169610, Singapore; 6 Office of Clinical Sciences, Duke-National University of Singapore Graduate Medical School, 8 College Rd, Singapore 169857, Singapore

**Keywords:** Yttrium-90 radioembolization, Selective internal radiation therapy, Partition model, Technetium-99m macroaggregated albumin SPECT/CT, CT lung densitovolumetry, Lung mass

## Abstract

**Background:**

For yttrium-90 (^90^Y) radioembolization, the common practice of assuming a standard 1,000-g lung mass for predictive dosimetry is fundamentally incongruent with the modern philosophy of personalized medicine. We recently developed a technique of personalized predictive lung dosimetry using technetium-99m (^99m^Tc) macroaggregated albumin (MAA) single photon emission computed tomography with integrated CT (SPECT/CT) of the lung as part of our routine dosimetric protocol for ^90^Y radioembolization. Its rationales are the technical superiority of SPECT/CT over planar scintigraphy, ease and convenience of lung auto-segmentation CT densitovolumetry, and dosimetric advantage of patient-specific lung parenchyma masses.

**Methods:**

This is a retrospective study of our pulmonary clinical outcomes and comparison of lung dosimetric accuracy and precision by ^99m^Tc MAA SPECT/CT versus conventional planar methodology. ^90^Y resin microspheres (SIR-Spheres) were used for radioembolization. Diagnostic CT densitovolumetry was used as a reference for lung parenchyma mass. Pulmonary outcomes were based on follow-up diagnostic CT chest or X-ray.

**Results:**

Thirty patients were analyzed. The mean lung parenchyma mass of our Southeast Asian cohort was 822 ± 103 g standard deviation (95% confidence interval 785 to 859 g). Patient-specific lung parenchyma mass estimation by CT densitovolumetry on ^99m^Tc MAA SPECT/CT is accurate (bias −21.7 g) and moderately precise (95% limits of agreement −194.6 to +151.2 g). Lung mean radiation absorbed doses calculated by ^99m^Tc MAA SPECT/CT and planar methodology are both accurate (bias <0.5 Gy), but ^99m^Tc MAA SPECT/CT offers better precision over planar methodology (95% limits of agreement −1.76 to +2.40 Gy versus −3.48 to +3.31 Gy, respectively). None developed radiomicrosphere pneumonitis when treated up to a lung mean radiation absorbed dose of 18 Gy at a median follow-up of 4.4 months.

**Conclusions:**

Personalized predictive lung dosimetry by ^99m^Tc MAA SPECT/CT is clinically feasible, safe, and more precise than conventional planar methodology for ^90^Y radioembolization radiation planning.

## Background

Radioembolization (RE) is intra-arterial brachytherapy using yttrium-90 (^90^Y) resin (SIR-Spheres®, Sirtex Medical Limited, North Sydney, New South Wales, Australia) or glass (TheraSphere®, BTG, Ottawa, Ontario, Canada) microspheres for the treatment of inoperable liver malignancies. Radiomicrosphere pneumonitis is a known complication due to hepatopulmonary shunting of ^90^Y microspheres from arteriovenous shunts within target hepatic arterial territories [[1]-[3]]. The severity of radiomicrosphere lung injury depends on the extent of hepatopulmonary shunting, the radiation absorbed dose, and its pulmonary biodistribution [[2],[4],[5]].

For ^90^Y resin microspheres, the ‘partition model’ is the simplest method of predictive dosimetry to personalize the intended radiation absorbed doses to tumor and non-tumorous liver and lung based on ^99m^Tc macroaggregated albumin (MAA) scintigraphy [[6],[7]]. For lung dosimetry, a standard mass of 1,000 g is often assumed [[8]] - an assumption which risks under- or overestimation of the lung radiation absorbed dose depending on patient size, pre-existing chronic lung disease, prior lung surgery or irradiation, lung shunt fraction (LSF), or injected ^90^Y activity. In the modern era of personalized medicine, there is a clinical need to shift away from assumed masses and to embrace patient-specific lung mass estimates for predictive dosimetry in ^90^Y RE.

For years, CT densitometry has been used by pulmonologists to evaluate lung parenchymal diseases such as emphysema [[9]]. Its premise is the approximate linear relationship between radiographic density, expressed in CT numbers (Hounsfield Unit, HU) and physical density (g/cm^3^) within the range of lung parenchyma [[10]]. This linear relationship was found to be consistent across a wide variety of CT scanners [[11]]. Hence, lung auto-segmentation CT densitovolumetry provides a simple, rapid, and convenient way of patient-specific lung parenchyma mass estimation.

We previously showed that ^99m^Tc MAA single photon emission computed tomography with integrated CT (SPECT/CT) of the liver can improve the safety and effectiveness of ^90^Y resin microsphere RE [[12]]. This is due its ability for attenuation and scatter correction of ^99m^Tc MAA activity and for volumetric assessment of target arterial territories to improve partition modeling. The next logical step was to expand ^99m^Tc MAA SPECT/CT dosimetry from the abdomen into the lung.

On the basis of the technical superiority of SPECT/CT over planar scintigraphy, ease and convenience of lung auto-segmentation CT densitovolumetry, and dosimetric advantage of using patient-specific lung parenchyma masses, we recently implemented ^99m^Tc MAA SPECT/CT of the lung into our routine dosimetric protocol, in addition to that of the abdomen [[12]]. This is a retrospective report of our clinical outcomes, with a detailed comparison of lung dosimetric accuracy and precision by SPECT/CT versus conventional planar methodology.

## Methods

Institutional review board approval was obtained for the conduct, waiver of informed consent, and publication of this retrospective study (CIRB 2010/781/C, SingHealth, Singapore). This study has been performed in accordance with the ethical standards laid down in the 1964 Declaration of Helsinki and its later amendments. Personalized predictive lung dosimetry by ^99m^Tc MAA SPECT/CT was recently integrated into our earlier protocol for liver dosimetry [[12]]. Our current protocol is now a single, fully SPECT/CT, seamless workflow from the lung to abdomen. For quality assurance of LSF calculation, conventional planar scintigraphy was also performed for each patient. ^90^Y resin microspheres (SIR-Spheres®, Sirtex Medical Limited, North Sydney, New South Wales, Australia) were used for RE.

Patients were eligible for this study if they have a pre-RE diagnostic CT chest and follow-up chest imaging at any time in the post-RE period. The follow-up chest imaging may either be a diagnostic CT or chest X-ray, and its purpose was to assess for any radiological features suggestive of radiomicrosphere pneumonitis. If both modalities were available, a diagnostic CT was favored over a chest X-ray. Patients enrolled in ongoing RE clinical trials were excluded.

### Imaging and reconstruction protocols

Pre-RE diagnostic CT chests were performed on six scanner types across our institution (Table 1). All diagnostic CT chests were acquired with inspiration and breath-holding, intravenous contrast, and tube voltage 120 kVp; milliamp seconds (mAs) varied widely. All images were reconstructed at 3-mm slice thickness, except for one scanner which was at 5 mm. Lung auto-segmentation was performed on images reconstructed using soft tissue kernels and 512 × 512 matrix; the images reconstructed using lung kernels were not used for auto-segmentation. Reconstructed fields-of-view ranged from 30 to 45 mm.

**Table 1:** Types of CT scanners

**Maker**	**Model**	**Number**
GE	LightSpeed VCT^a^	11
Philips	iCT 256	4
Siemens	SOMATOM Definition	8
Siemens	SOMATOM Sensation	1
Toshiba	Aquilion	4
Toshiba	Aquilion ONE	2

^a^Reconstructed at 5-mm slice thickness.

All scintigraphy was performed on a Philips Precedence SPECT/CT scanner (Philips Healthcare, Eindhoven, The Netherlands), which has a dual-head gamma camera integrated with a 16-slice multi-detector CT. All scintigraphy were performed within 1 h of ^99m^Tc MAA injection. For planar imaging, anterior and posterior images of the lung and liver were acquired over 120 s each and matrix size 256 × 256. Attenuation and scatter correction was not performed for planar images.

For SPECT acquisition, 128 frames (20 s per frame, angle step of 3°) were acquired over 360° with a 128 × 128 matrix using a low-energy, high-resolution collimator at photopeak 140 ± 10% keV. All patients were imaged from the lung apex to inferior liver edge, covering one or two bed positions depending on the combined lengths of the lung and liver. SPECT images were reconstructed using Astonish software (Philips Healthcare, Eindhoven, The Netherlands), which is an iterative 3D-ordered subset expectation maximization (3D-OSEM) algorithm incorporating corrections for resolution recovery, attenuation, and scatter correction based on the CT attenuation map, three iterations eight subsets and no filters. Non-contrast-enhanced CT was acquired at free breathing at 120 kVp and 50 mAs. CT images were reconstructed using a soft tissue kernel at 3-mm slice thickness, reconstructed field-of-view 60 cm and matrix size 512 × 512.

### Phantom evaluation of CT densitometry

An approximately linear relationship between CT numbers and physical density has been shown to be valid for CT numbers ≤0 HU [[11],[13]], where 0 HU is the CT number of water. For quality assurance, CT densitometry was performed on a 1-L water phantom scanned on our SPECT/CT using the same CT settings as for ^99m^Tc MAA SPECT/CT. Its results were compared to an expected water physical density of 1 g/cm^3^. A large volume-of-interest (VOI) was drawn within the water phantom to obtain its mean CT number, and the physical density (g/cm^3^) was calculated as [[10],[14],[15]]:

(1) $$\mathrm{Physical}\;\mathrm{density}\;=\;\frac{\mathrm{Mean}\;\mathrm{CT}\;\mathrm{number}\;+\;1000}{1000}$$

As we had no access to suitable lung phantoms, our CT numbers closer to that of lung density were not assessed. For the purposes of this study, it was assumed that the CT phantom results reported by Cheng et al. [[11]] were applicable to our SPECT/CT scanner.

### Lung CT densitovolumetry

Lung CT densitovolumetry for both diagnostic CT and SPECT/CT was performed by auto-segmentation using OsiriX software version 5.6 (Pixmeo, Geneva, Switzerland). The aim was to obtain a *reasonable estimate* of the patient-specific lung parenchyma mass for dosimetry, conceptually superior to the standard 1,000 g assumption. It was not our aim to obtain a patient's true lung parenchyma mass, which remains unknown. Lung parenchyma masses derived by diagnostic CT were used as a reference for comparison against that derived by SPECT/CT.

On diagnostic CT, the entire left and right lung parenchyma were auto-segmented slice by slice from apex to costophrenic recesses using a two-dimensional trans-axial growing algorithm. The lower threshold CT number was fixed at −1,000 HU, while the upper threshold was visually adjusted between −150 and −600 HU to obtain the best segmentation result for each slice. Each trans-axial region-of-interest (ROI) was visually reviewed and manually refined if necessary. The intent was to include all lung parenchyma and to exclude large hilar vessels, proximal bronchial tree, trachea, and large parenchymal scars, in accordance with external beam radiotherapy conventions [[16]]. Trans-axial ROIs for the left and right lungs were interpolated separately to compute their respective volumes and mean CT numbers. The latter was applied into Equation 1 to calculate the approximate lung mean densities (g/cm^3^) for the left and right lungs. The patient-specific lung parenchyma mass was calculated from the product of its volume and lung mean density for both the left and right lungs.

**Figure 1:**
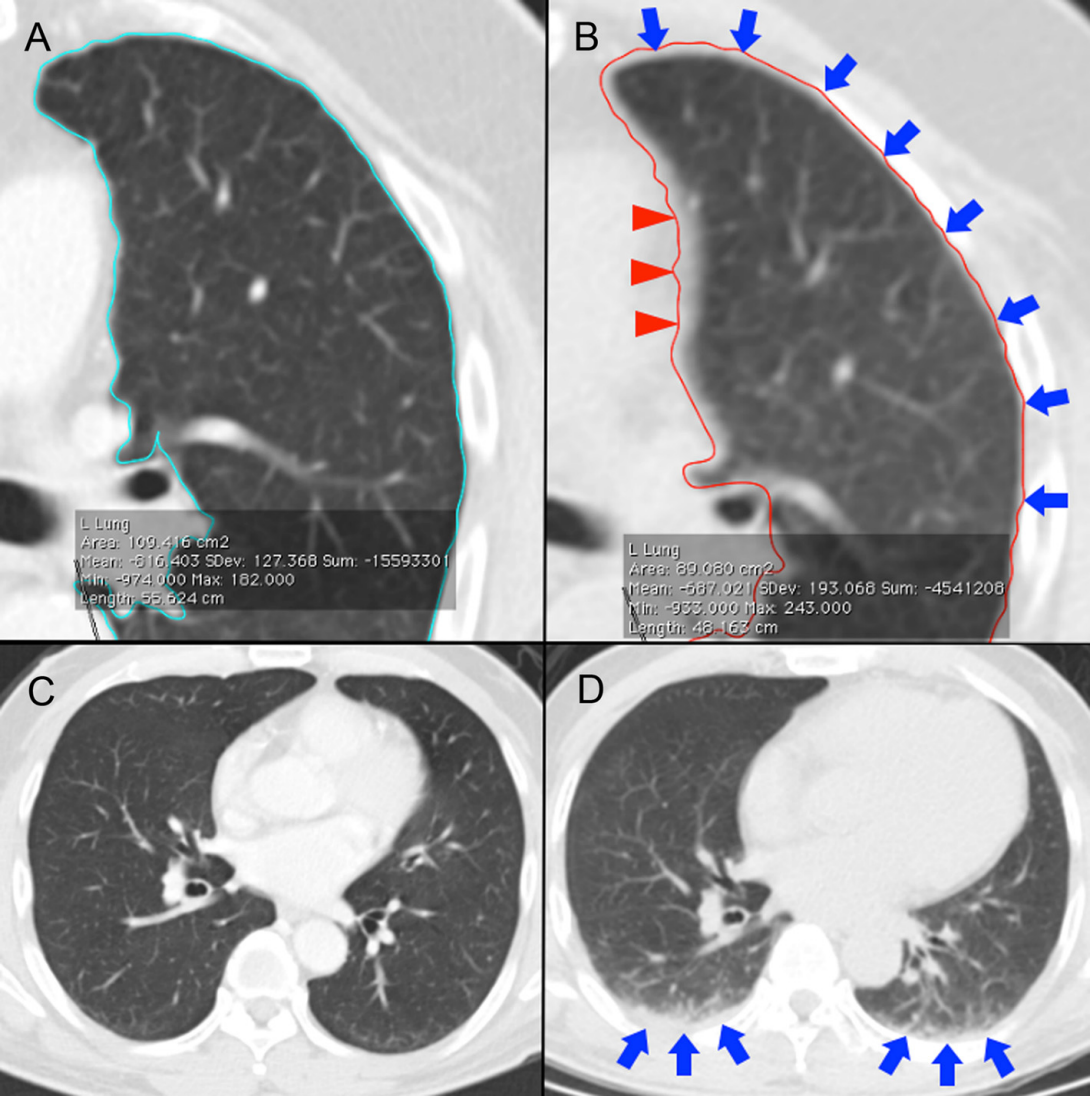
**Technical considerations in region-of-interest (ROI) delineation.** On diagnostic CT, auto-segmented ROIs should closely match lung parenchyma margins **(A)**. Our SPECT/CT ROI technique requires the inclusion of a very thin sliver of pleura within its margins (blue arrows), but this has a tendency to over-include the left mediastinal border (red triangles) **(B)**. Due to differences in CT scan technique, diagnostic CT **(C)** and SPECT/CT **(D)** of the same patient performed a week apart show dependent atelectasis on SPECT/CT (**D**, blue arrows) but not on diagnostic CT **(C)**.

On SPECT/CT, lung CT densitovolumetry was performed in a similar manner but with a slight difference to ROI margins. Prior to the implementation of our current dosimetric protocol, we conducted an internal pilot study to assess the technical feasibility of lung CT densitovolumetry by SPECT/CT. We observed that for SPECT/CT to obtain lung parenchyma mass estimates comparable to that by diagnostic CT for the same patient, the SPECT/CT ROI margins should be slightly more generous than that of diagnostic CT. This is achieved by including a thin sliver of pleura within its margins not exceeding 1 mm in thickness (Figure 1), which we believe to be related to differences in lung expansion between an inspiratory breath-hold diagnostic CT versus free-breathing SPECT/CT. We also observed that dependent atelectasis was more common in SPECT/CT (Figure 1). This was likely due to a combination of free-breathing CT acquisition and prolonged lying in the supine position from exploratory angiography to SPECT/CT. Our SPECT/CT ROIs were adjusted to include all regions of dependent atelectasis. At our institution, an experienced operator can complete the entire lung segmentation process within 20 min, including manual refinements.

### Lung shunt fraction calculation

LSFs for both planar scintigraphy and SPECT/CT were calculated by conventional formularism [[3],[8]] and expressed as percentages:

(2) $$\mathrm{LSF}\;=\;\frac{\mathrm{Total}\;\mathrm{lung}\;\mathrm{counts}}{\mathrm{Total}\;\mathrm{lung}\;\mathrm{counts}\;+\;\mathrm{Total}\;\mathrm{counts}\;\mathrm{within}\;\mathrm{all}\;\mathrm{target}\;\mathrm{hepatic}\;\mathrm{arterial}\;\mathrm{territories}}$$

Planar image analysis was performed using Philips Extended Brilliance Workspace Nuclear Medicine software version 2.0 (Philips Healthcare, Eindhoven, The Netherlands). Planar ROIs were contoured over the left and right lungs and the liver, excluding the mediastinum. Simple background activity correction was performed using a method similar to that described by Jha et al. [[17]]. Scatter correction was not performed to be consistent with conventional methodology [[3],[8]]. Both anterior and posterior images were contoured to obtain geometric means of lung and liver counts.

**Figure 2:**
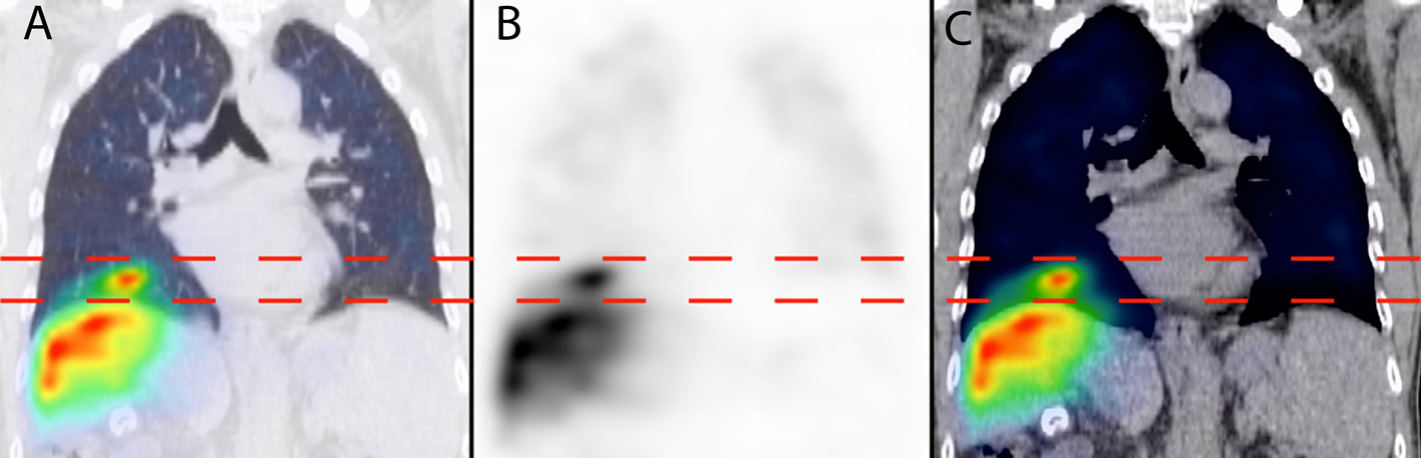
**Example of**^**99m**^**Tc MAA SPECT/CT mis-registration at the right diaphragm due to free breathing.** This is depicted in coronal views of SPECT/CT lung window **(A)**, SPECT **(B)**, and SPECT/CT soft tissue window **(C)**. Horizontal dashed lines indicate the mis-registration extent.

SPECT/CT acquired at free breathing is known to cause mis-registration of ^99m^Tc MAA activity from the liver dome into both lung bases (Figure 2), overestimating the LSF [[18]]. To overcome this problem, Yu et al. recently proposed using either the left lung only or to exclude the lung regions near the diaphragms [[18]]. Neither solution is ideal, as the pulmonary biodistribution of ^90^Y microspheres may be asymmetrical between both sides of the lung and also within each lung. We chose the latter method of excluding the lung regions near the diaphragms for our protocol.

**Figure 3:**
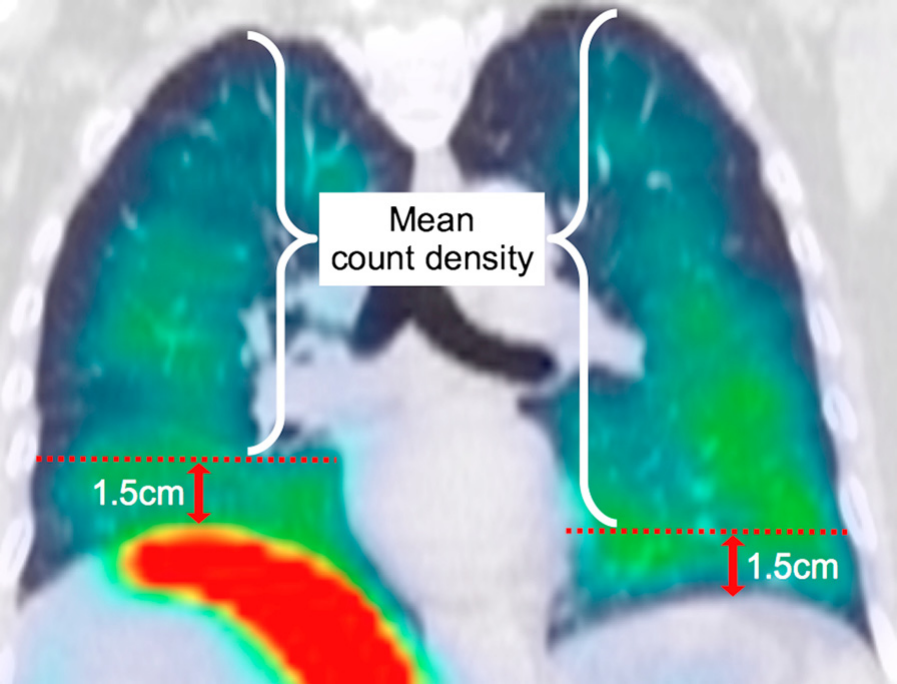
**Overview of lung count quantification.** Horizontal dashed lines indicate the trans-axial cut-off levels for bilateral exclusion zones, 1.5 cm above each diaphragm apex. Background-corrected total lung counts were calculated from the lung mean count density above the exclusion zones.

The cranio-caudal displacement of the diaphragm apex during free breathing was measured by Kolar et al. to be 2.73 ± 1.02 cm (mean ± standard deviation) [[19]]. We empirically chose a figure of 1.5 cm above the apex of both diaphragms as the trans-axial cut-off level, below which all lung SPECT counts were excluded from all dosimetric analysis, i.e., ‘exclusion zone’ (Figure 3). Depending on the relative positions of the diaphragm apices at the time of CT, the exclusion zone cut-offs for both lungs may not always be at the same trans-axial level (Figure 3).

Due to variable *in vivo* breakdown of ^99m^Tc MAA, background activity may be present due to free pertechnetate within the blood pool and soft tissue, in addition to scatter. On SPECT/CT, simple lung background activity correction was performed by first estimating the background count density (counts/cm^3^) using a long cylindrical VOI placed along the left erector spinae muscle in the posterior abdomen. The lung background counts above the exclusion zone were calculated as the product of the background count density and the lung parenchyma tissue volume above the exclusion zone. The lung parenchyma *tissue* volume (not to be confused with CT lung volume) was estimated as follows: lung parenchyma mass above exclusion zone/1.04, where 1.04 g/cm^3^ is the assumed physical density of general soft tissue (not be confused with lung mean density estimated from Equation 1). The lung background counts above the exclusion zone were subtracted accordingly to obtain the background-corrected lung counts above the exclusion zone. This was further divided by the CT lung volume above the exclusion zone to obtain the background-corrected lung mean count density (counts/cm^3^), which we assumed to be representative of the entire lung. The background-corrected counts for the entire lung were calculated as the product of the background-corrected lung mean count density and the total CT lung volume on SPECT/CT. To complete the LSF denominator of Equation 2, background-corrected counts of all target hepatic arterial territories were obtained as previously described for liver SPECT/CT dosimetry [[12]].

### Lung mean absorbed dose calculation

By Medical Internal Radiation Dose (MIRD) formularism, the lung mean radiation absorbed dose, *D*_mean_, expressed in gray (Gy), was calculated as follows:

(3) $$\mathrm{Lung}\;\mathrm{mean}\;\mathrm{dose}=\;\frac{49.67\times \mathrm{LSF}\times \mathrm{Total}\;\mathrm{injected}\;\mathrm{activity}}{\mathrm{Lung}\;\mathrm{parenchyma}\;\mathrm{mass}}$$

where 49.67 is the absorbed dose coefficient of 1 GBq of ^90^Y uniformly distributed throughout 1 kg of tissue [[6]], LSF is in its original dimensionless ratio, activity in GBq and mass in kilogram. ^90^Y resin microspheres were used, and all cases were planned by partition modeling [[12]]. Our dosimetric limit for the intended lung mean dose is 25 Gy for a single RE [[8]]. The use of SPECT-based voxel dosimetry to derive dose-volume histograms was not explored in this study.

For the purposes of this study, the ‘reference *D*_mean_’ (i.e., gold standard) for each patient was assumed to be best approximated by a combination of SPECT/CT LSF and lung parenchyma mass derived by diagnostic CT densitovolumetry. ‘Planar methodology’ refers to conventional *D*_mean_ calculation based on planar LSF and the standard 1,000 g assumption. ‘SPECT/CT methodology’ refers to personalized *D*_mean_ calculation using SPECT/CT to derive both the LSF and the patient-specific lung parenchyma mass.

### Statistical analysis

Data were assumed to follow a normal distribution and presented in mean ± standard deviation (SD), median, and 95% confidence intervals (CI). Paired two-tailed *t* test was used to compare the difference between the means of two related datasets; a *p* value <0.05 was considered statistically significant. Bland-Altman methodology was used to compare two methods of measurement, where the mean difference between the two methods is the bias and represents *accuracy*, while the 95% limits of agreement (LOA) is the bias ± 1.96 SD and represents *precision*.

## Results

A total of 63 consecutive patients underwent RE over 1 year and 4 months. Of these, 30 patients were eligible for analysis. Table 2 summarizes the patient, disease, treatment, and follow-up characteristics of our cohort. One patient had mild emphysema evident on diagnostic CT chest; all others were normal. The highest *D*_mean_ in this cohort was 18 Gy (Table 3); all underwent RE once. At a median follow-up of 4.4 months, none showed evidence of radiomicrosphere pneumonitis on follow-up chest imaging.

**Table 2:** **Patient characteristics, injected**^**90**^**Y activity, and follow-up chest imaging**

	**Values**^**a**^
Gender	
Male	24
Female	6
Ethnicity	
Chinese	20
Indonesian	5
Myanmar	4
Others	1
Liver malignancy	
Hepatocellular carcinoma	25
Metastatic colon cancer	4
Cholangiocarcinoma	1
Age (years)^b^	
62.5 ± 11.2; 63.5	58.5 to 66.5; 28 to 80^c^
Injected 90Y activity (GBq)^b^	
1.65 ± 0.78; 1.44	1.37 to 1.93; 0.50 to 3.62^c^
Time from pre-RE diagnostic CT chest to 99mTc MAA SPECT/CT (weeks)^b^	
10.4 ± 21.8; 1.9	2.6 to 18.2; 0.3 to 105.3^c^
Time to follow-up chest imaging (months)^b^	
4.9 ± 3.4; 4.4	3.7 to 6.1; 1.2 to 17.5^c^
Follow-up chest imaging modality	
Diagnostic CT chest	25
Chest X-ray	5

^a^Values are expressed as *n* unless otherwise specified; ^b^mean ± SD; median values; ^c^95% CI; range values.

**Table 3:** Lung quantification results

**Item**	**Mean ± SD; median; 95%CI; range**	***t*** **test**	**Bias ± SD; 95%LOA**
Lung mean density (g/cm^3^)			
Diagnostic CT			
Right lung	0.215 ± 0.049; 0.201; 0.197 to 0.232; 0.121 to 0.376		
Left lung, compared to right	0.217 ± 0.054; 0.201; 0.197 to 0.236; 0.128 to 0.383	*p* = 0.44	+0.0019 ± 0.0131; −0.0239 to +0.0276
Mean of both lungs	0.216 ± 0.051; 0.206; 0.197 to 0.234; 0.125 to 0.379		
SPECT/CT			
Right lung	0.291 ± 0.051; 0.289; 0.273 to 0.309; 0.180 to 0.410		
Left lung, compared to right	0.306 ± 0.064; 0.310; 0.273 to 0.309; 0.161 to 0.442	*p* = 0.002	+0.0152 ± 0.0243; −0.0324 to +0.0628
Mean of both lungs, compared to diagnostic CT	0.299 ± 0.056; 0.298; 0.278 to 0.319; 0.171 to 0.417	*p* <0.001	
Lung parenchyma mass (g)			
Diagnostic CT			
All patients	822 ± 103; 809; 785 to 859; 621 to 1,107		
Male	828 ± 114; 823; 783 to 874; 621 to 1,107		
Female	795 ± 19; 801; 780 to 811; 764 to 812		
SPECT/CT			
All patients, compared to diagnostic CT	788 ± 110; 736; 748 to 827; 624 to 1,045	*p* = 0.19	−21.7 ± 88.2; −194.6 to +151.2
Lung volume (cm^3^)			
Diagnostic CT	3,967 ± 798; 4,049; 3,682 to 4,253; 2,142 to 5,634		
SPECT/CT, compared to diagnostic CT	2,749 ± 744; 2,530; 483 to 3,015; 1,698 to 5,199	*p* <0.001	
Lung shunt fraction (%)			
SPECT/CT	5.96 ± 4.59; 4.97; 4.32 to 7.61; 0.98 to 21.71		
Planar, compared to SPECT/CT	7.36 ± 4.96; 6.03; 5.58 to 9.14; 2.20 to 25.26	*p* <0.001	+1.40 ± 1.60; −1.73 to +4.52
Lung *D*_mean_ (Gy)			
Reference *D*_mean_	6.16 ± 5.24; 3.64; 4.28 to 8.03; 0.76 to 17.52		
Planar, compared to reference	6.07 ± 4.51; 4.75; 4.46 to 7.68; 1.00 to 16.02	*p* = 0.78	−0.09 ± 1.73; −3.48 to +3.31
SPECT/CT, compared to reference	6.48 ± 5.51; 4.06; 4.51 to 8.45; 0.85 to 18.87	*p* = 0.11	+0.32 ± 1.06; −1.76 to +2.40
Planar, compared to SPECT/CT		*p* = 0.27	−0.41 ± 2.00; −4.32 to +3.50

### Technical assurance

Water phantom studies obtained a mean CT number of water of 8.8712 ± 3.3887 HU (range −6 to 25 HU, VOI 518 cm^3^). By Equation 1, the calculated physical density of water was 1.0089 ± 0.0034 g/cm^3^ (range 0.9940 to 1.025). Compared to an expected water density of 1 g/cm^3^, this result shows a low mean error of +0.9 ± 0.3% for CT densitometry on our SPECT/CT system.

On diagnostic CT, the mean lung mean density was 0.216 ± 0.051 g/cm^3^ (95% CI 0.197 to 0.234). There was no statistically significant difference between the means of the left versus right lung mean densities (*p* = 0.44). Bland-Altman analysis showed both the bias and 95% LOA between the left and right lung mean densities to be clinically insignificant (Table 3). Overall, these results provide technical assurance that lung CT densitometry was accurate, precise, and repeatable.

### SPECT/CT densitovolumetry

**Figure 4:**
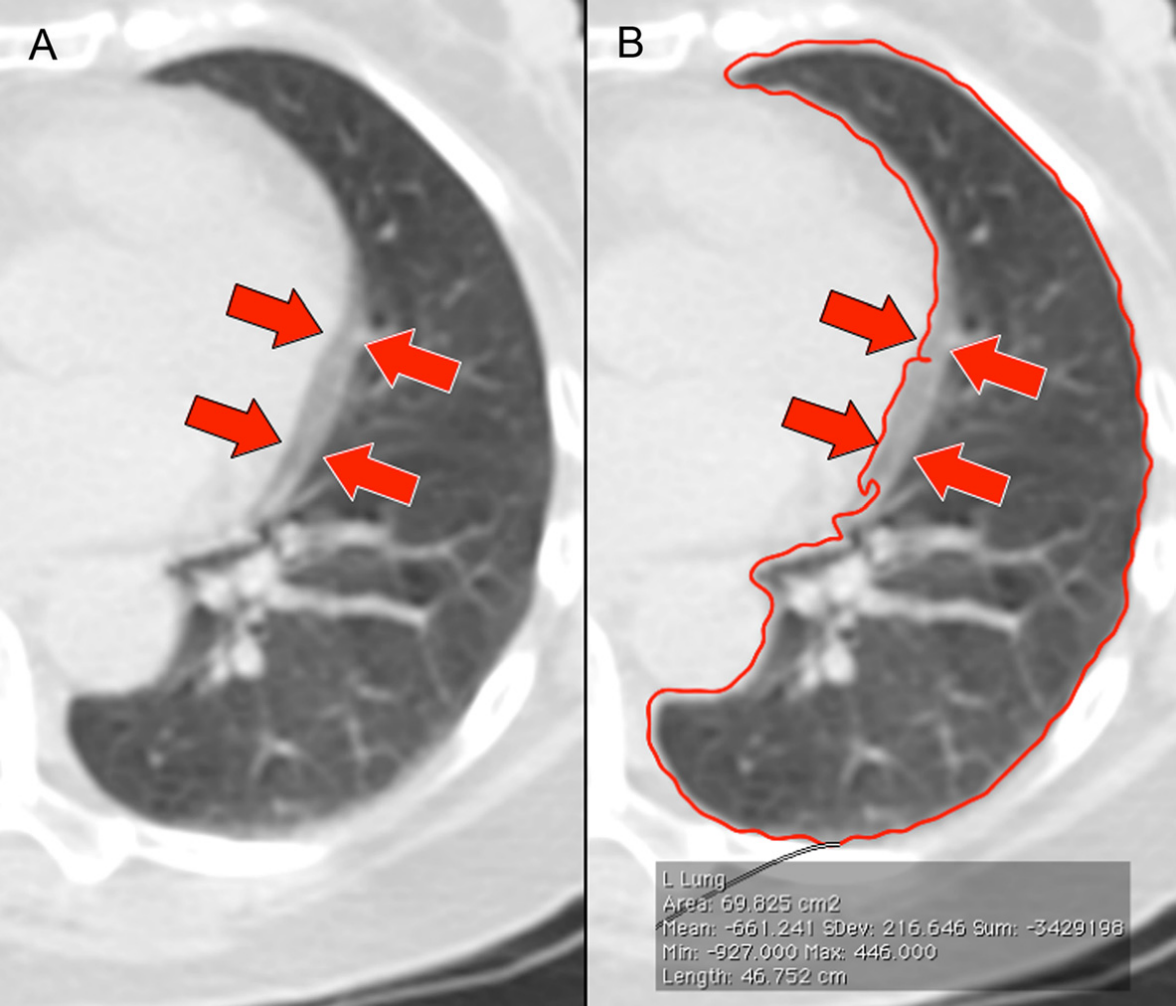
**Cardiac motion artefacts on SPECT/CT.** Cardiac motion artefacts were frequently observed at the left mediastinal border (arrows) **(A)**, often leading to auto-segmentation errors (red contour line) **(B)** requiring manual correction.

On SPECT/CT, the mean lung mean density was 0.299 ± 0.056 g/cm^3^ (95% CI 0.278 to 0.319). We found a small but statistically significant difference between the means of the left and right lung mean densities (*p* = 0.002). This was probably caused by cardiac motion artefacts at the left mediastinal border affecting the left lung auto-segmentation, incompletely corrected by manual refinement (Figure 4). However, Bland-Altman analysis showed the bias to be clinically insignificant (Table 3).

**Figure 5:**
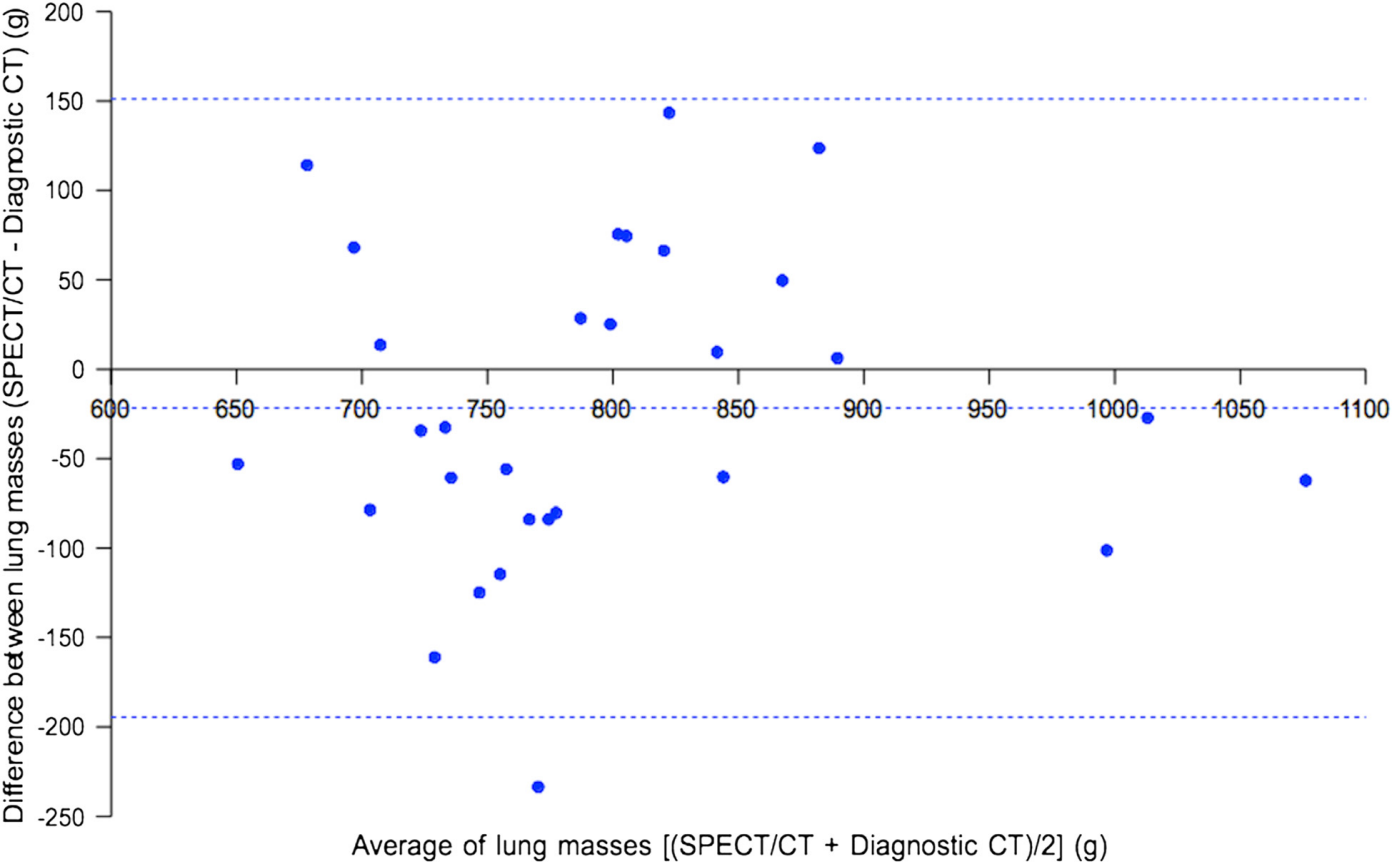
**Bland-Altman plot of lung CT densitovolumetry parenchyma masses by SPECT/CT versus diagnostic CT.** Horizontal dashed lines indicate the bias and 95% LOA.

By diagnostic CT, the mean lung parenchyma mass of our Southeast Asian cohort was 822 ± 103 g (95% CI 785 to 859). There was no statistically significant difference in the mean lung parenchyma mass by SPECT/CT compared to diagnostic CT (*p* = 0.19). Bland-Altman analysis (Figure 5) showed SPECT/CT to have a small bias of −21.7 g, which was dosimetrically insignificant. However, its 95% LOA was moderately large, ranging from −194.6 to +151.2 g (Table 3). Overall, these results show that our SPECT/CT technique of lung parenchyma mass estimation was accurate but only moderately precise.

### Lung volume

The mean lung volume by SPECT/CT was significantly smaller (*p* <0.001) than that by diagnostic CT (Table 3). This was an expected finding because SPECT/CT was acquired at free breathing while diagnostic CT was acquired at inspiratory breath-holding.

### Lung shunt fraction

**Figure 6:**
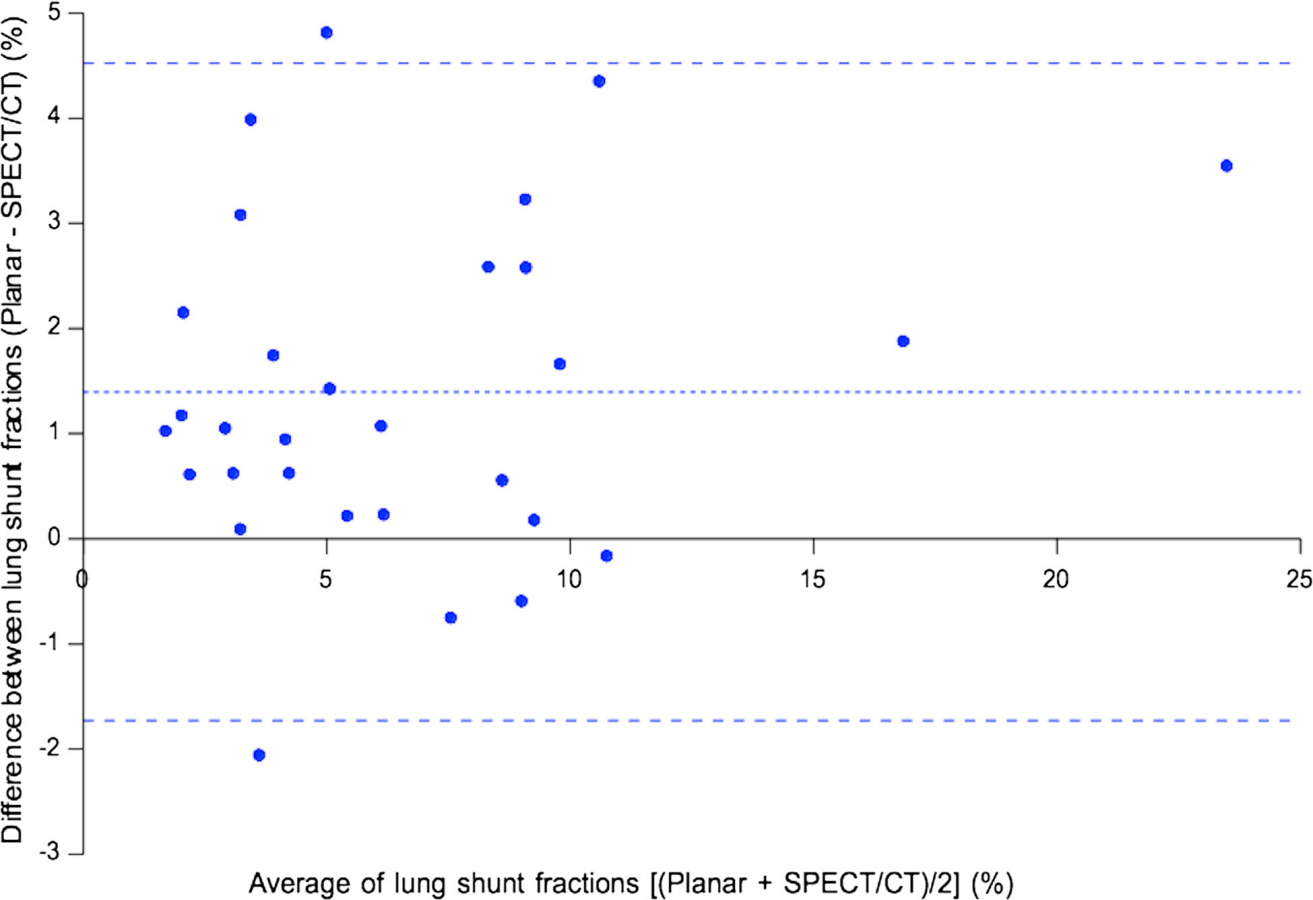
**Bland-Altman plot of LSFs by planar scintigraphy versus SPECT/CT.** Horizontal dashed lines indicate the bias and 95% LOA.

There was a small but statistically significant difference between the mean LSFs by planar scintigraphy and SPECT/CT (*p* <0.001). Bland-Altman analysis (Figure 6) showed planar LSFs to have a small bias of +1.4% compared to SPECT/CT (Table 3).

### Lung mean absorbed dose

**Figure 7:**
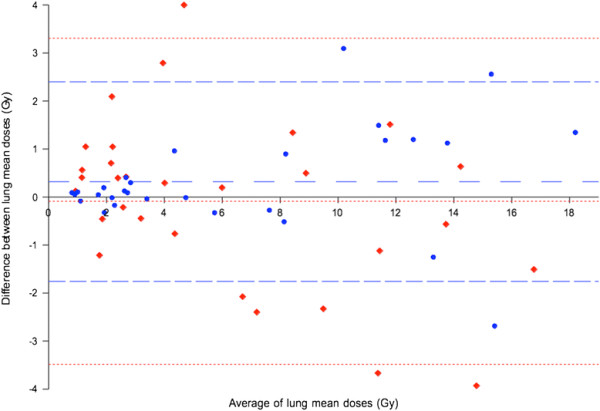
**Combined Bland-Altman plots of lung mean doses by planar and SPECT/CT methodologies.** Planar (red diamonds) and SPECT/CT (blue circles) methodologies were each respectively compared to the reference *D*_mean_. Fine red and coarse blue horizontal dashed lines indicate the bias and 95% LOA for planar and SPECT/CT methodologies, respectively.

To reiterate, the reference *D*_mean_ (i.e., gold standard) for each patient was assumed to be best approximated by a combination of SPECT/CT LSF and lung parenchyma mass derived by diagnostic CT densitovolumetry. By this definition, we found no statistically significant differences between the mean *D*_mean_ estimated by either planar or SPECT/CT methodology, when each was respectively compared to the mean reference *D*_mean_ (*p* >0.05). Bland-Altman analysis (Figure 7) showed both planar and SPECT/CT methodologies to have biases of <0.5 Gy when each was respectively compared to reference *D*_mean_, which is dosimetrically acceptable. However, 95% LOA for SPECT/CT was smaller than that for planar methodology (95% LOA −1.76 to +2.40 Gy versus −3.48 to +3.31 Gy, respectively) (Table 3). Overall, these results show that lung dosimetry by planar and SPECT/CT methodologies are both accurate, but SPECT/CT offers better precision. The results also show the small but statistically significant LSF over-estimation by planar scintigraphy to be dosimetrically insignificant in the context of *D*_mean_.

When the mean *D*_mean_ by planar methodology was directly compared to that by SPECT/CT methodology, we found no statistically significant difference between the two methods (*p* = 0.27). Bland-Altman analysis showed planar methodology to have a small bias of −0.41 Gy compared to SPECT/CT, which was dosimetrically insignificant. However, its 95% LOA was moderately large, ranging between −4.32 and +3.5 Gy. Overall, these results suggest moderate disagreement between planar and SPECT/CT methodologies for *D*_mean_ calculation and, therefore, should not be used interchangeably (Table 3).

## Discussion

Safe and effective partition modeling relies on the accurate input of dosimetric parameters such as tissue masses, tumor-to-normal liver ratio and LSF. We previously showed that ^99m^Tc MAA SPECT/CT of the abdomen can improve partition modeling for the liver [[12]]. As an extension of our earlier work, this study clinically validates ^99m^Tc MAA SPECT/CT for personalized predictive lung dosimetry and has provided initial data on its safety, accuracy, and precision.

Our lung CT densitovolumetry results are in keeping with the literature. Rosenblum et al. found the whole-lung mean CT number among 19 subjects under inspiratory breath-holding to be −802 ± 34 HU and among 44 subjects under free breathing to be −734 ± 58 HU [[14]]. These figures translate into a mean lung mean density of 0.198 ± 0.034 g/cm^3^ (95% CI 0.182 to 0.214) by inspiratory breath-hold CT and 0.266 ± 0.058 g/cm^3^ (95% CI 0.249 to 0.283) by free-breathing CT, comparable to our results. Our study has also found planar LSFs to be slightly overestimated as compared to SPECT/CT LSFs. This observation was consistent with the literature and may be explained by the lack of attenuation and scatter correction in planar scintigraphy [[18],[20]-[22]].

Our study has shown conventional planar methodology, with its assumption of a standard 1,000 g lung mass, to be dosimetrically accurate, affirming its strong history of clinical safety. However, we found planar methodology to be less precise than SPECT/CT methodology. While our data shows planar methodology to be accurate when analyzed across a cohort, its relative imprecision risks dosimetric uncertainty for patients who fall beyond population norms. Examples are patients with extremes of height, pre-existing chronic lung disease (e.g., emphysema, interstitial lung disease), undergone prior lung surgery or irradiation. Such patients should not be assumed to have ‘standard’ lung masses or lung parenchymal radiobiology and will benefit from personalized predictive lung dosimetry. Its clinical impact is greatest under the paradigm of partition modeling, which relies heavily on dosimetric accuracy and precision for the safe escalation of intended tumor absorbed doses. Furthermore, we have found the mean lung parenchyma mass of our Southeast Asian cohort to be nearly always less than 1,000 g. Ultimately, planar methodology is undesirable from a dosimetric perspective because its assumption of a standard 1,000 g lung mass is fundamentally incongruent with the modern era of personalized medicine.

We found planar methodology to have a dosimetrically insignificant bias of −0.41 Gy compared to SPECT/CT methodology for *D*_mean_ calculation. However, its 95% LOA was moderately large (−4.32 to +3.5 Gy) and therefore these two methods cannot be regarded as dosimetrically equivalent. This means that a choice of either method would not be expected to significantly affect lung dosimetry in patients of average anthropometry but may significantly impact those who fall beyond population norms. Although correlations exist across a population between lung parenchyma mass and physical parameters such as age, body weight, or height, modern personalized medicine encourages clinicians to pursue patient-specific measurements for individualized therapeutic guidance. Therefore, we advocate SPECT/CT methodology to be routinely used for lung predictive dosimetry and advise against using planar and SPECT/CT methodologies interchangeably for *D*_mean_ calculation.

SPECT/CT methodology overcomes most of the technical limitations of planar methodology and also enables CT densitovolumetry for patient-specific lung parenchymal mass estimation. On the technical issue of SPECT/CT mis-registration due to free breathing, this problem similarly affects planar scintigraphy and therefore should not be regarded as a comparative disadvantage. On the contrary, SPECT/CT can partially overcome this problem using the technique outlined in this report. Respiratory-gated ^99m^Tc MAA SPECT/CT is a promising solution which warrants investigation.

Although our SPECT/CT technique for patient-specific lung parenchyma mass estimation was shown to be accurate, it was only moderately precise. Lung CT densitovolumetry may be affected by many physiological and technical factors such as the phase of respiration [[14]], variations in pulmonary blood flow [[15]], intravenous contrast, CT reconstruction [[23]], and ROI delineation methods. Further research is necessary to improve its precision on SPECT/CT. However, it is important to reiterate that our aim was not to derive a patient's true lung parenchyma mass, which remains unknown, but its reasonable estimate. To this end, we believe our SPECT/CT methodology has served its purpose well.

A limitation of our study was the lack of a true gold standard for *D*_mean_ for clinical validation of our SPECT/CT methodology. ^99m^Tc MAA is an imperfect surrogate for ^90^Y resin microspheres due to slightly dissimilar physical properties [[24]]. It is also theoretically possible for LSFs to dynamically change during RE due to progressive microembolization. Therefore, LSFs simulated by ^99m^Tc MAA only give an estimate of the true post-RE LSF. The relative contribution to errors in *D*_mean_ due to biophysical and technical inaccuracies from ^99m^Tc MAA and scintigraphic methods are beyond the scope of this study, However, we believe ^99m^Tc MAA SPECT/CT to be feasible for personalized predictive lung dosimetry until superseded by better simulation microspheres (e.g., fluorine-18 resin microspheres [[25]]) or imaging modalities (e.g., respiratory-gated ^90^Y PET/CT [[26]]). Another limitation of our study was the assumption of normal distribution for our data for statistical tests of significance. We believe this assumption to be reasonably valid for our sample size of 30 patients.

We have clinically validated our SPECT/CT methodology up to *D*_mean_ 18 Gy without radiomicrosphere pulmonary toxicity. This study has also provided early data on the statistical limits of dosimetric uncertainty for *D*_mean_ planned by ^99m^Tc MAA, represented by the bias and LOA. This statistical data may guide personalized predictive lung dosimetry in a manner similar to that recently described for the liver [[27]].

## Conclusions

Patient-specific lung parenchyma mass estimation by CT densitovolumetry on ^99m^Tc MAA SPECT/CT is accurate and moderately precise. Lung mean radiation absorbed doses calculated by ^99m^Tc MAA SPECT/CT is accurate and offers better precision than planar methodology. An integrated, seamless, and personalized dosimetric workflow by ^99m^Tc MAA SPECT/CT from lung to abdomen is clinically feasible and safe.

## Competing interests

YHK, KHT, PKHC, and ASWG received research funding from Sirtex Medical Singapore. PKHC and ASWG receive honoraria from Sirtex Medical Singapore. This study was partially funded by a research grant from Sirtex Medical Singapore.

## Authors' contributions

YHK, BMM, YT, ASWG, and DCEN were involved in study design, implementation, data collection, data analysis, and manuscript preparation. KHT and PKHC were involved in radioembolization, clinical care, and manuscript preparation. All authors read and approved the final manuscript.
